# Epidrugs: Toward Understanding and Treating Diverse Diseases

**DOI:** 10.3390/epigenomes6030018

**Published:** 2022-07-12

**Authors:** Takashi Umehara

**Affiliations:** Laboratory for Epigenetics Drug Discovery, RIKEN Center for Biosystems Dynamics Research, 1-7-22 Suehiro-cho, Tsurumi, Yokohama 230-0045, Japan; takashi.umehara@riken.jp; Tel.: +81-45-503-9457

**Keywords:** chromatin, DNA, epigenetics, histone, nucleosome, post-translational modification

## Abstract

Epigenomic modifications are unique in the type and amount of chemical modification at each chromosomal location, can vary from cell to cell, and can be externally modulated by small molecules. In recent years, genome-wide epigenomic modifications have been revealed, and rapid progress has been made in the identification of proteins responsible for epigenomic modifications and in the development of compounds that regulate them. This Special Issue on “Epidrugs: Toward Understanding and Treating Diverse Diseases” aims to provide insights into various aspects of the biology and development of epigenome-regulating compounds.

The epigenome forms, in many of its parts, a basic compacted structure, the nucleosome, consisting of the histone octamer (two copies each of H2A, H2B, H3, and H4) and 145 to 147 base pairs of DNA [1,2]. The major epigenomic modifications include acetylation, methylation, and phosphorylation of the side chains of residues in the N-terminal tails of the histones [3,4]. Another important epigenomic modification is the methylation of cytosine bases in the CpG sequence of DNA [5,6]. In epigenomic regulation, there are often three types of actions for each chemical modification: (1) writing, (2) reading, and (3) erasing [4,7]. The proteins responsible for each action usually contain a cavity to recognize the small epigenetic modifications (such as acetylation or methylation), often allowing their structural and functional control by small molecules, a critical feature for epidrug development.

Several epidrugs that target histone or DNA modifications have already been developed as therapeutic agents for refractory cancers. For example, some histone deacetylase inhibitors, such as vorinostat (SAHA) and romidepsin (FK228), are therapeutic agents for cutaneous T-cell lymphoma [8,9]. In addition, tazemetostat, an inhibitor of the histone methyltransferase EZH2, was recently approved by the United States Food and Drug Administration (FDA) for the treatment of follicular lymphoma [10,11]. The nucleoside analogues 5-azacytidine and decitabine are known drugs that inhibit DNA methyltransferases (DNMT) in the treatment of myelodysplastic syndromes [12,13]. Nucleoside non-analogues that selectively inhibit DNMT1 [14,15] have also recently been of interest for the treatment of acute myeloid leukemia.

Epidrugs and related chemical probes are expected to be useful not only for disease therapy but also for elucidating the basic functions of the epigenome. For example, trapoxin, a histone deacetylase inhibitor, led to the cloning of the first histone deacetylase gene by the ligand affinity method [16]. Epidrugs have also been useful in analyzing the dynamics of modification in the histone proteome [17] and for developing the chem-seq method to identify the genome-wide location of a compound bound to a protein of interest [18]. In addition, the “bump-and-hole” strategy, in which a target protein can be orthogonally regulated by a compound, has been realized for BET proteins involved in recognizing histone acetylation [19]. Furthermore, inhibitors of BET proteins have been used in studies to degrade target proteins using PROTAC (proteolysis targeting chimera) [20,21,22], leading to the postulation that PROTAC is a promising strategy for future epidrug development.

Finally, epidrug development to date has primarily focused on refractory cancers. Indeed, aberrant gene expression in cancer may be regulated by the positive feedback of epigenomic modification and its recognition [23,24], and epidrugs may suppress many intractable cancers in addition to those mentioned above. Furthermore, clinical trials of epidrugs are increasingly targeting diseases other than cancer, which may lead to the use of epidrugs against a wide variety of diseases in the future.
